# App-Based Training Module on Guiding Physicians’ Prescription for Antibiotic Treatment of Gonorrhea: Cluster Randomized Controlled Trial

**DOI:** 10.2196/63736

**Published:** 2026-03-04

**Authors:** Ting-Ting Jiang, Xiao-Bin Zhang, Pei-Zhen Zhao, Li-Juan Fei, Hui-Min Chen, Yun-Qing Yang, Man-Hong Jia, Cheng Wang, Yun-Liang Shen, Yu-Jun Xu, Yan Han, Yue-Ping Yin, Xiang-Sheng Chen

**Affiliations:** 1 Hospital for Skin Diseases, Institute of Dermatology, Chinese Academy of Medical Sciences & Peking Union Medical College, 12 Jiangwangmiao Street, Nanjing, 210042, China, 86 2585478901; 2 Yunnan Center for Disease Control and Prevention, Kunming, China; 3 Dermatology Hospital, Southern Medical University, Guangzhou, China; 4 Zhejiang Provincial Institute of Dermatology, Huzhou, China; 5 Hainan Fifth People's Hospital, Haikou, China; 6 Guangzhou Dermatology Hospital, Guangzhou, China

**Keywords:** gonorrhea, prescribing, treatment guideline, nonadherence, randomized controlled trial, survey, online training, mobile app, interview

## Abstract

**Background:**

The Chinese National Guidelines on Diagnosis and Treatment of Gonorrhea (2020) recommend ceftriaxone 1 g intramuscularly as a single dose for the treatment of uncomplicated gonococcal infections. However, nonadherence to the guidelines remains common among physicians in China, partly due to their poor awareness of the recommendations.

**Objective:**

This trial aimed to (1) assess the effectiveness of an app-based training tool in improving adherence to guideline-recommended treatment regimens and (2) identify reasons for nonadherence among physicians who used the training tool.

**Methods:**

Using a cluster randomized controlled trial design, we randomly allocated 72 hospitals (clusters) from 4 provinces to the intervention (n=36, 50%) or control (n=36, 50%) arms in a 1:1 ratio. In the intervention arm, physicians received free app-based training via smartphones for 6 months, while the control arm continued routine training. The primary outcome was the proportion of prescriptions adherent to the regimens for treatment of uncomplicated gonorrhea recommended by the national guidelines. We analyzed cluster-level summary outcomes at endline using methods appropriate for stratified cluster randomized controlled trials with relatively few clusters per group, accounting for between-cluster variation. A postintervention survey was administered to all intervention-arm physicians to evaluate their experience with the app-based training module. Additionally, face-to-face interviews were conducted with 32 purposely selected nonadherent physicians from the intervention arm to explore reasons for nonadherence.

**Results:**

A total of 715 physicians (n=343, 48% in the control arm and n=372, 52% in the intervention arm) participated in the study. Over the 6-month intervention period, the adherence to 1 g ceftriaxone for the treatment of uncomplicated gonorrhea increased from 53.6% to 54.8% in the intervention arm, whereas the rate decreased from 43.9% to 42.5% in the control arm. The mean difference (5.5%, 95% CI −7% to 18%; *P*=.37) in changes in the adherence rate between intervention and control arms was not statistically significant (risk ratio 1.12, 95% CI 0.93‐1.35; *P*=.23). Among the 32 (19%) nonadherent physicians from the intervention arm, the primary reason for nonadherence was their concern regarding the sufficiency of the currently recommended dosage of generic ceftriaxone for treating gonorrhea.

**Conclusions:**

In our study, the implementation of an app-based training tool did not improve adherence to treatment recommendations. Concern regarding the efficacy of generic ceftriaxone may be one of the intrinsic factors associated with nonadherence.

## Introduction

Gonorrhea, caused by *Neisseria gonorrhoeae*, is a significant global public health issue, with an estimated 82.4 million cases among adults aged 15 to 49 years in 2020 [1]. Gonococcal infection predominantly involves the epithelium of the urethra, endocervix, rectum, oropharynx, and conjunctivae and can also ascend to the upper genital tract to cause pelvic inflammatory disease in women and epididymo-orchitis in men [2]. Effective control of gonorrhea relies on prevention, accurate diagnostics, and appropriate antimicrobial treatment. However, the treatment of gonorrhea is complicated by the ability of *N gonorrhoeae* to develop resistance to antimicrobials [3,4].

Due to the widespread emergence of resistant strains, only ceftriaxone is now recommended as the first-line regimen for treatment of gonorrhea in most countries [5,6]. However, isolates with decreased susceptibility to ceftriaxone, the last remaining option for first-line empiric treatment of gonorrhea, have been identified in Japan [7], the United States [8], France [9], Spain [10], the United Kingdom [11], and Australia [12]. In China, the percentage of *N gonorrhoeae* isolates with decreased susceptibility to ceftriaxone ranged between 9.7% and 12.2% from 2013 to 2016 [13].

In response to the growing threat of antimicrobial resistance, the 2020 European gonorrhea guidelines recommended high-dose ceftriaxone (1 g) for uncomplicated gonorrhea when antimicrobial susceptibility is unknown [5]. Similarly, the 2021 US Centers for Disease Control and Prevention sexually transmitted infections treatment guidelines recommended ceftriaxone 500 mg for individuals weighing <150 kg and 1 g for those weighing ≥150 kg [6]. In 2020, Chinese experts also reached a consensus to increase the ceftriaxone dose for uncomplicated gonorrhea to 1 g [14].

A major driver of antimicrobial resistance is the inappropriate use of antibiotics, particularly overprescribing, which can lead to the selection of resistant organisms. A nationwide study in China revealed a high proportion of prescription behaviors that were nonadherent to the national guidelines on diagnosis and treatment of gonorrhea in China (hereinafter referred to as the National Guidelines) [15], with excessive ceftriaxone dosing being common [16]. Lack of awareness of the National Guidelines was identified as a key factor contributing to nonadherence [15].

To address this issue, we developed a smartphone app–based training tool aimed at improving guideline adherence among physicians in China. We conducted a cluster randomized controlled trial to assess its effectiveness on improving adherence to the guidelines (ie, prescribing ceftriaxone 1 g intramuscular as a single dose for treatment of uncomplicated gonococcal infections). The secondary aim was to explore the reasons for nonadherence among physicians who used the training tool.

## Methods

### Ethical Considerations

This study was registered at the Chinese Clinical Trial Registry [17] (ChiCTR2000029591, registered on February 5, 2020) and approved by the Medical Ethics Committee of the Chinese Academy of Medical Sciences Institute of Dermatology (2020-LS-004). This study adhered to the principles of the Declaration of Helsinki. Participation in the study was voluntary and anonymous, and informed consent was obtained from all participants. No compensation was provided to participants.

### Study Design and Setting

We conducted a parallel-group, cluster randomized controlled trial in 72 hospitals in 4 provinces (Guangdong, Hainan, Yunnan, and Zhejiang) in China to compare the adherence rate for treating uncomplicated gonorrhea with 1 g ceftriaxone between the intervention (app-based training module) and control groups.

Provinces were selected based on the reported incidence of gonorrhea in 2018, with high-incidence areas prioritized. Within each province, city-level hospitals with high reported gonorrhea cases were invited. After obtaining written consent from hospital directors, baseline data (eg, hospital category, gonorrhea case volume, physician qualifications, and drug availability) were collected. Hospitals were stratified by province and randomly assigned to intervention or control arms using a computer-generated sequence in R software (version 3.5.3; R Foundation for Statistical Computing), with allocation concealed until assignment. An internal pilot was conducted in 18 hospitals (n=9, 50% intervention and n=9, 50% control) in Guangdong province to assess feasibility. After confirming acceptability (166/241, 68.9% of physicians trained), the trial expanded to all 72 hospitals (n=36, 50% per arm), with no protocol changes between phases.

### Participant and Intervention

The study was conducted from March 2022 to February 2023. In Guangdong, the 6-month intervention ran from March to August 2022, followed by a questionnaire survey in intervention hospitals in September. For Hainan and Yunnan, the intervention was implemented from April to September 2022, with the survey conducted in October. In Zhejiang, the intervention period was August 2022 to January 2023, followed by the survey in February 2023.

In China, medical graduates must complete a mandatory 3-year standardized residency training program following their undergraduate medical education (typically a 5-year program). This nationally regulated system, implemented through accredited teaching hospitals, ensures uniform clinical competency across all medical practitioners. For positions in major metropolitan hospitals (particularly tier-1 cities), competitive candidates generally require additional postgraduate qualifications beyond the mandatory training. All physicians working in dermatology, urology, andrology, gynecology, and venereology departments at participating hospitals were eligible for study inclusion, irrespective of their educational background. After providing informed consent, physicians were guided by the local study coordinator to download the Xieshou app [18], an official platform developed by the National Center for STD Control of the Chinese Center for Disease Control and Prevention. The Xieshou platform is an electronic system routinely used for sexually transmitted disease (STD) health education and partner management in hospitals and is managed by STD control institutions at various levels. Health facilities are encouraged to integrate this platform into routine STD services. Through the platform, physicians can direct patients to scan a QR code to automatically receive an electronic intervention package containing health education materials, condom promotion information, mobilization for testing, and treatment instructions. Additionally, physicians can use the platform for academic exchanges, experience sharing, and participation in training sessions and conferences (including training videos, case discussions, and conference notices).

Technicians of the Xieshou platform distinguished between intervention and control group physicians based on hospital names provided during registration. In intervention hospitals, physicians were prompted to complete an online questionnaire collecting background information (sex, age, education level, years of practice, working department, professional title, previous training, and number of gonorrhea cases treated). After submission, they received unlimited access to a training video on the National Guidelines for the treatment of gonorrhea. Over the following 6 months, study coordinators sent monthly WeChat (Tencent Holdings Limited) reminders to physicians to watch the video, which was automatically saved in their personal learning records. At the end of the intervention, physicians in these hospitals were invited to complete another online questionnaire (Multimedia Appendix 1) via the Xieshou app to provide feedback on current treatment recommendations and their experience with the intervention. In control group hospitals, physicians were only prompted to complete the baseline questionnaire and did not have access to the training video after submission.

### Outcomes

The primary outcome was the proportion of prescriptions adherent to the regimens for treatment of uncomplicated gonorrhea recommended by the National Guidelines at the cluster level. The secondary outcome was the reasons for nonadherence among physicians in the intervention arm.

### Data Collection and Analyses

In each hospital, physicians’ prescription records were extracted from the Hospital Information System using keyword matching (ie, gonorrhea). Prescriptions for uncomplicated gonorrhea from 6 months before (baseline) and 6 months after (endline) trial initiation were assessed for adherence to treatment guidelines. Prescriptions were excluded from data analysis if they were prescribed to patients who met any of the following criteria: (1) younger than 18 years; (2) were pregnant or lactating women; (3) received no ceftriaxone; (4) were treated with antibiotics for other infections; or (5) were diagnosed as complicated gonorrhea, such as disseminated gonococcal infection and pelvic inflammatory disease. Patient demographic data, gonorrhea diagnosis, and treatment regimens (antibiotic selection and dosage) were collected. To measure the adherence rate to the National Guidelines, a single 1 g dose of ceftriaxone was considered in line with the guidelines; deviations were classified as nonadherence. Two researchers independently reviewed the prescriptions and made the adjudication, and any discrepancies were discussed until agreement was reached.

For the secondary outcome, we purposively sampled 1 physician per intervention hospital (with ≥25% nonadherent prescriptions at endline). A total of 32 in-depth, face-to-face interviews were conducted in Chinese by local coordinators using a semistructured interview outline (Multimedia Appendix 2). Interview topics focused on intervention experiences, perspectives on antibiotic prescribing for gonorrhea treatment, and clinical practices. Interviews were audio-recorded upon written consent, and notes were taken. Participants were given the option to withdraw from the study at any point without any consequences. All interviews occurred in quiet and private locations in local hospitals.

### Statistical Analysis

On the basis of a nationwide survey in China [15], we estimated that the adherence rate to the National Guidelines for the treatment of uncomplicated gonorrhea would be 37.8%, and that we could collect and process an average of 100 prescriptions per hospital. Clinical experts deemed a 10% increase in adherence clinically meaningful for the intervention arm. Under the assumption of a coefficient of variation of 0.1, 10% data loss due to illegible prescriptions, and accounting for stratification, the study required 144 clusters to detect this increase with 90% power at a 5% significance level (2-sided test). Eight provinces (Jiangsu, Shanghai, Zhejiang, Fujian, Guangdong, Guangxi, Hainan, and Yunnan) with the highest incidence of reported gonorrhea cases in China were selected in our study protocol [19]. Within each province, 9 hospitals were planned for the control group and 9 for the intervention group. However, the target number of hospitals (n=144) was not met due to COVID-19 pandemic–related restrictions in Jiangsu, Shanghai, Fujian, and Guangxi.

Participating hospital and physician characteristics were summarized using frequencies (and sample sizes) and means (SDs) as appropriate for each group. For the primary outcome, we used analysis methods appropriate for stratified, cluster-randomized trials with relatively few clusters per arm to account for between-cluster variation. We pooled both pilot and main trial data for our analysis of prescriptions, which was based on the original allocation of hospitals (ie, on an intention-to-treat basis). To deal with skewness in the distribution of cluster outcomes, we first log-transformed the proportions. We then estimated the overall risk ratio based on a weighted average of stratum-specific risk ratios, which were calculated as the difference between intervention and control arm log risks (equivalent to ratios of geometric mean risks between treatment arms), with the weights inversely proportional to stratum-specific variances. We then estimated an approximate SE for the log risk ratio and used this to calculate 95% CIs for the risk ratio and carry out formal hypothesis testing (2 sided and at the 5% level) via a stratified 2-tailed *t* test. To adjust for covariates, we fitted a logistic regression model to the individual-level data with terms for the covariates of interest, excluding the treatment arm, and then calculated cluster-specific ratio residuals based on the ratio of observed to model-predicted values. Results were adjusted for stratum (province), cluster-level outcome at baseline, patient’s sex and age, and physician’s sex and department. We also calculated risk difference results using similar methods to those used to calculate the risk ratio results. In addition, we explored the associations between nonadherence and physician characteristics (age, sex, education level, years of practice, working department, professional title, number of treated gonorrhea cases, and previous training) without the intervention and added another exploratory outcome after protocol development: a physician-level indicator of nonadherence in the nonintervention group. Variables that showed significant association with the nonadherence (*P*<.05) in univariate analyses were included in the multivariate regression model to explore the association of variables with the outcome. All statistical analyses were conducted using SPSS Statistics for Windows (version 22.0; IBM Corp).

### Qualitative Data Analysis

The qualitative interviews were recorded, deidentified, transcribed in Chinese, and then translated into English for analysis. The transcribed data were thematically analyzed in NVivo 11 (Lumivero). Two researchers trained in qualitative analysis methods, TTJ and YQY, analyzed several transcripts to identify themes and create codes and subcodes, which were organized into categories. This coding framework was applied to all transcripts by both researchers and modified through an iterative process.

## Results

### Baseline Characteristics

A total of 68 hospitals (n=33, 48.5% in the control group and n=35, 51.5% in the intervention group) provided background information. Overall, baseline hospital characteristics were well balanced between the 2 groups (Table 1). A total of 715 physicians (n=343, 48% in the control group and n=372, 52% in the intervention group) completed the baseline questionnaire survey. The intervention group had a higher proportion (n=233, 62.6%) of female physicians and included more physicians (n=213, 57.2%) from the STD department and the department of obstetrics and gynecology (Table 2).

**Table 1.** Baseline characteristics of participating hospitals.

Characteristics	Control (n=33)	Intervention (n=35)
Hospital nature, n (%)		
General hospital	29 (87.9)	28 (80.0)
Specialized hospital	4 (12.1)	7 (20.0)
Hospital township, n (%)		
Public teaching hospital	27 (81.8)	27 (77.1)
Public nonteaching hospital	6 (18.2)	7 (20.0)
Hospital category, n (%)		
Tertiary hospital	24 (72.7)	21 (60.0)
Secondary hospital	8 (24.2)	10 (28.6)
Number of gonorrhea cases reported in 2019, mean (SD)	124 (21)	146 (25)
Number of physicians qualified for managing patients with sexually transmitted disease, mean (SD)	33 (4)	25 (3)
Hospital had been stocked with ceftriaxone, n (%)		
Yes	32 (97.0)	33 (94.3)
No	1 (3.0)	2 (5.7)
Hospital had been stocked with spectinomycin, n (%)		
Yes	14 (42.4)	16 (45.7)
No	19 (57.6)	19 (54.3)

**Table 2.** Baseline characteristics of physicians.

Characteristics	Control (n=343), n (%)	Intervention (n=372), n (%)
Sex		
Male	157 (45.8)	139 (37.4)
Female	186 (54.2)	233 (62.6)
Age group (years)		
<35	128 (37.3)	131 (35.2)
35-44	122 (35.6)	123 (33.1)
45-55	70 (20.4)	87 (23.4)
>55	23 (6.7)	31 (8.3)
Highest degree		
Postgraduate and above	128 (37.3)	130 (34.9)
Undergraduate	204 (59.5)	224 (60.2)
Junior college and below	11 (3.2)	18 (4.8)
Working department		
Dermatology	62 (18.1)	68 (18.3)
Obstetrics and gynecology	87 (25.4)	134 (36.0)
Urology	74 (21.6)	60 (16.1)
Andrology	12 (3.5)	6 (1.6)
Sexually transmitted disease	35 (10.2)	79 (21.2)
Service (years)		
≤5	88 (25.7)	88 (23.7)
6-10	73 (21.3)	72 (19.4)
≥11	182 (53.1)	212 (57.0)
Professional title		
Chief physician	43 (12.5)	43 (11.6)
Associate chief physician	94 (27.4)	117 (31.5)
Physician-in-charge	132 (38.5)	122 (32.8)
Resident physician	69 (20.1)	69 (18.5)
Number of cases of uncomplicated gonorrhea treated in the last 3 months		
≤1	293 (85.4)	296 (79.6)
2-4	44 (12.8)	69 (18.5)
≥5	6 (1.7)	7 (1.9)
Having received relevant training on treatment of gonorrhea in the last 3 months		
Yes	217 (63.3)	214 (57.5)
No	126 (36.7)	158 (42.5)

### Nonadherence to the National Guidelines

Among the 1524 prescriptions (n=747, 49% prescriptions in the control group and n=777, 51% baseline prescriptions in the intervention group) with physician demographic data, 734 (48.2%) were nonadherent to the National Guidelines for ceftriaxone treatment of uncomplicated gonorrhea.

In the multivariate analyses, the following factors were found to be significantly associated with nonadherence after adjusting for potential confounding factors: age <35 years (adjusted odds ratio [AOR] 2.61, 95% CI 1.74‐3.92, compared with ≥35 years; *P*<.001); undergraduate degree and below (AOR 2.63, 95% CI 1.99‐3.47, compared with postgraduate degree and above; *P*<.001), working in a department of dermatology (AOR 4.05, 95% CI 2.93‐5.58, compared with the department of STD; *P*<.001); and treating fewer gonorrhea cases (<5 cases) in the past 3 months (AOR 2.66, 95% CI 1.67‐4.23, compared with treating ≥5 cases; *P*<.001; Table 3).

**Table 3.** Factors associated with nonadherence to the treatment guidelines in the control group^a^.

Characteristics	Nonadherence (n=734), n (%)	Adherence (n=790), n (%)	AOR^b^ (95% CI)
Sex			
Male	475 (64.7)	606 (76.7)	Reference
Female	259 (35.3)	184 (23.3)	1.21 (0.87-1.67)
Age group (years)			
<35	194 (26.4)	122 (15.4)	2.61 (1.74-3.92)^c^
≥35	540 (73.6)	668 (84.6)	Reference
Highest degree			
Undergraduate and below	572 (77.9)	512 (64.8)	2.63 (1.99-3.47)^c^
Postgraduate and above	162 (22.1)	278 (35.2)	Reference
Working department			
Dermatology	320 (43.6)	132 (16.7)	4.05 (2.93-5.58)^c^
Obstetrics and gynecology	35 (4.8)	50 (6.3)	1.14 (0.66-1.98)
Urology	226 (30.8)	385 (48.7)	1.25 (0.90-1.74)
Andrology	6 (0.8)	39 (4.9)	0.31 (0.12-0.79)
Sexually transmitted disease	120 (16.3)	184 (23.3)	Reference
Professional title			
Resident physician	96 (13.1)	65 (8.2)	1.30 (0.72-2.36)
Physician-in-charge	307 (41.8)	269 (34.1)	1.24 (0.85-1.83)
Associate chief physician	249 (33.9)	254 (32.2)	1.54 (1.07-2.21)
Chief physician	82 (11.2)	202 (25.6)	Reference
Number of treated cases of gonorrhea in the last 3 months			
<5	701 (95.5)	673 (85.2)	2.66 (1.67-4.23)^c^
≥5	33 (4.5)	117 (14.8)	Reference

a Variables including age, sex, education level, working department, professional title, and number of treated cases of gonorrhea in the last 3 months were included in the multivariate analysis.

b AOR: adjusted odds ratio.

c *P*<.001.

### Impact on Improvement of Adherence

**Figure 1.**
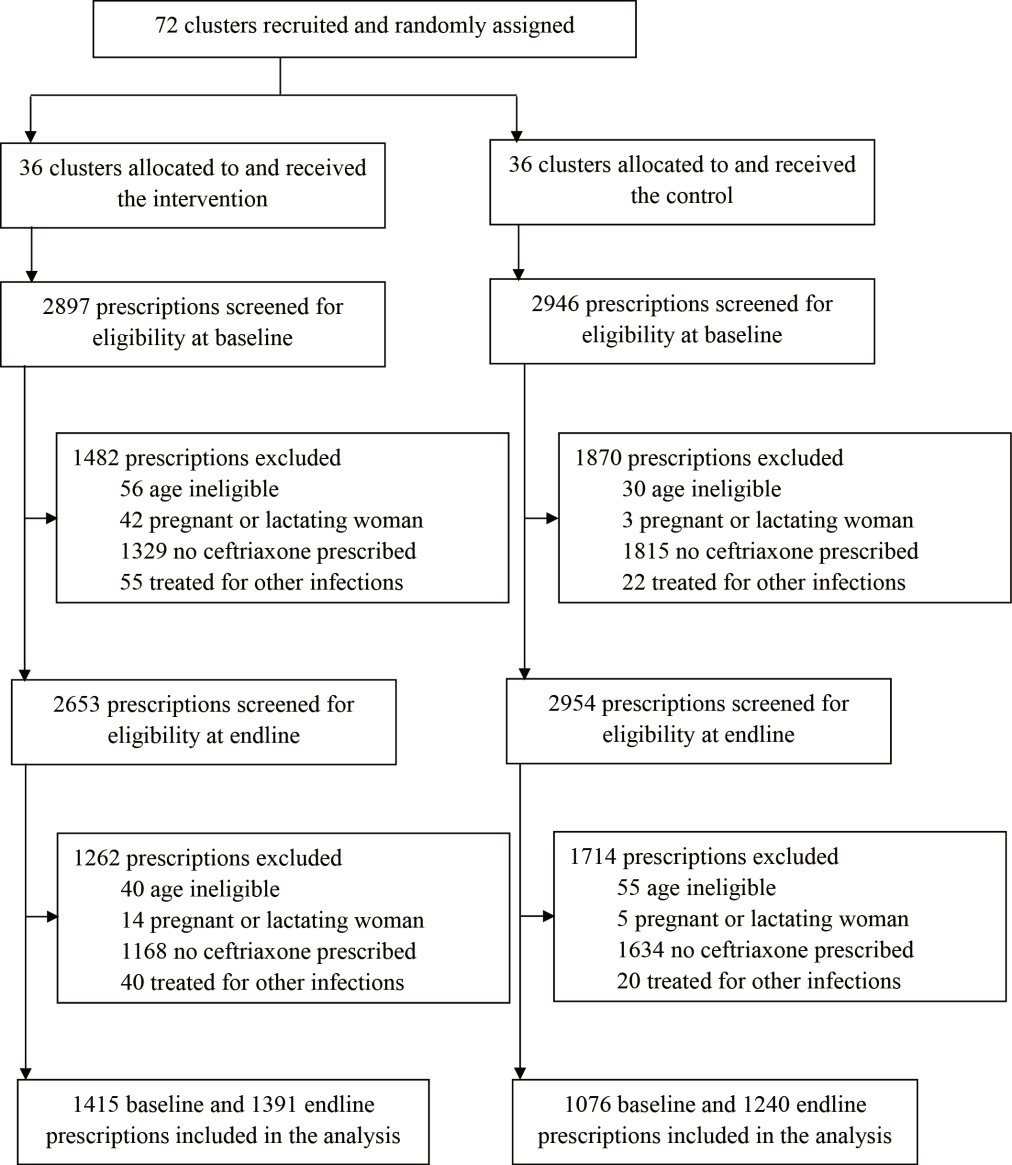
Trial profile. Clusters refer to hospitals. Baseline prescriptions were issued during the 6 months before the implementation of the intervention, whereas endline prescriptions were issued during the 6-month intervention period.

Of the 3254 patients diagnosed with gonorrhea at baseline, 2171 (66.7%) were treated with ceftriaxone, whereas among the 3191 patients diagnosed with gonorrhea at endline, 2137 (67%) underwent treatment with ceftriaxone. We screened 2897 baseline and 2653 endline prescriptions from the intervention group, of which 1415 (48.8%) baseline prescriptions and 1391 (52.4%) endline prescriptions were eligible, respectively, for inclusion in the analysis (Figure 1). In the control group, 36.5% (1076/2946) of baseline and 42% (1240/2954) of endline prescriptions were eligible (Figure 1). The adherence rate increased from 53.6% to 54.8% in the intervention group, while the rate decreased from 43.9% to 42.5% in the control group. We also noted the variation of the adherence rate in the intervention clusters between baseline and endline was from −67% to 71% (mean difference 1.3%, 95% CI −8.5% to 11.0%; *P*=.79, paired *t* test), whereas in the control group it ranged from −50% to 100% (mean difference −1.4%, 95% CI −11.5% to 8.7%; *P*=.78, paired *t* test). After controlling for potential confounders, the online training intervention was not significantly associated with changes in the adherence rate (mean difference 5.5%, 95% CI −7% to 18%; *P*=.37; risk ratio 1.12, 95% CI 0.93 to 1.35; *P*=.23). However, when further comparing the compliance trend between the control and intervention groups, differences in trends of mean compliance of prescriptions were observed (*P*<.001; Multimedia Appendix 3). In addition, when the comparison was done by province, an obvious increase in mean compliance of prescriptions could be observed in Yunnan, where baseline compliance was substantially low (Multimedia Appendix 3).

### Reasons for Nonadherence to the National Guidelines

A total of 64 physicians in the intervention group completed the questionnaire assessing their experience with the app-based training. Most respondents (n=54, 84.4%) reported having completed the monthly training, and 9 (14.1%) reported being unaware of the latest National Guidelines before the intervention.

In the intervention group, 168 physicians did not adhere to the National Guidelines. To investigate reasons for nonadherence, 32 (19%) physicians were interviewed. Concerns about the efficacy of generic ceftriaxone emerged as the predominant theme across all interviews:

We are all using generic ceftriaxone. I typically opt for a 2 g dose due to concerns regarding the efficacy of generic ceftriaxone.

[A dermatologist]

In China, the national essential medicines scheme and zero-markup policy have been instrumental in severing the connection between prescribing practices and hospital revenues. However, these policies also restrict the availability of certain antibiotics:

*We perceive that the efficacy of generic drugs is inferior to that of branded drugs. There appears to be a reduced selection of drugs available compared to before, with many effective branded drugs no longer accessible. The remaining generic drugs seem to be of lower quality*.

[A gynecologist]

Instances have been reported in which generic antibiotics failed to effectively treat gonorrhea, further eroding physicians’ confidence in generics:

*I believe that the dosage and duration of generic ceftriaxone 1 g single dose for treating gonorrhea are inadequate. Patients treated with this regimen still experienced purulent discharge from the urethral orifice on the second and third days post-treatment, leading to a higher recurrence rate*.

[A urologist]

## Discussion

### Principal Findings

In this study, we used an electronic platform to deliver training on gonorrhea treatment guidelines to physicians in hospital settings. The results demonstrated that the online training module did not significantly improve physician adherence to the National Guidelines for treating uncomplicated gonorrhea with ceftriaxone (*P*=.23). However, the intervention showed a more pronounced effect in Yunnan province, where adherence in the intervention group increased by 15%. This notable improvement may be attributed to the region’s exceptionally low baseline adherence rate (4%) compared with the pooled baseline adherence rate of other provinces (65%). In contrast, areas with moderate to high baseline adherence rates (Zhejiang: 49%; Guangdong: 72%; and Hainan: 77%) showed no significant behavioral changes in the postintervention period, suggesting that nonadherence in these regions was unlikely due to a lack of guideline awareness. This hypothesis is further supported by the finding that adherence rates did not differ significantly between physicians who were aware of the updated National Guidelines (26/55, 47.3%) and those who were not (5/9, 55.6%) in the intervention group. Postintervention interviews among physicians who were not adherent to the guidelines revealed that concerns about the efficacy of generic ceftriaxone were the primary reason for deviating from the guidelines.

### Comparison With Prior Work

Ceftriaxone is a broad-spectrum bactericidal agent that belongs to the third-generation cephalosporins [20]. It was patented, manufactured, and marketed in 1982 as Rocephin (Roche Pharmaceuticals). Following the expiration of the Rocephin patent, generic products became available in many countries. Compared with the brand-name (innovator) product, generics significantly reduce health care costs and enhance supply security due to their diverse sources [21,22]. Consequently, Asian markets are dominated by low-cost, locally or regionally manufactured generic medicines [23], leading to a wide availability of generic ceftriaxone for gonorrhea treatment. The efficacy and safety of these generics are theoretically assured by bioequivalence studies, making them interchangeable with innovator products [24]. However, numerous studies have found that generic ceftriaxone products often fail to meet the pharmaceutical quality standards of the branded original [25-27]. A survey of the quality of generic ceftriaxone products from Eastern Asia markets revealed high levels of impurities and microbiologic contamination, raising significant efficacy and safety concerns [28]. Physicians in this study reported treatment failures with generics in clinical practice, which may have led them to prescribe excessive doses of ceftriaxone. Overuse of antibiotics, prescription of branded antibiotics, and prescription of antibiotics not listed in the National List of Essential Medicines are prevalent in other limited-income countries [29]. Physician perceptions that branded drugs are superior to generics may contribute to both the overuse of generics and the preferential prescription of branded alternatives [29,30].

In China, ceftriaxone susceptibility testing is conducted using the innovator product rather than generics. It remains unclear whether a 1 g single dose of generic ceftriaxone is adequate for uncomplicated gonorrhea due to the lack of postmarketing surveillance and follow-up studies in China. Notably, 15.6% (10/64) of physicians in this study still considered the 1 g dose insufficient. Thus, further research is needed to generate evidence on the efficacy of currently available generic antibiotics for gonorrhea and to strengthen surveillance of resistance to these generics.

Although evidence-based guidelines have been developed, translating knowledge into practice is challenging. One potential reason for the intervention’s limited success is the 6-month duration, which may have been insufficient to alter prescribing behavior. Time-trend analyses in this study demonstrated that extending the intervention period led to upward trends in appropriate antibiotic use for gonorrhea. Other studies have similarly found that discontinuing effective behavioral interventions resulted in a resurgence of inappropriate prescribing [31]. Additionally, online training alone may have a limited impact on reducing antibiotic misuse. A multifaceted approach is crucial for success. Interventions such as physician education, computerized clinical decision support, audit and feedback, punishment, and financial incentives, when implemented in combination, have resulted in lower rates of antibiotic misuse in other settings [32,33].

### Strengths and Limitations

To our knowledge, this study represents the first cluster randomized controlled trial conducted in China to evaluate the impact of an innovative training program on reducing physicians’ nonadherence to gonorrhea treatment guidelines.

However, several limitations should be considered when interpreting the findings. First, the COVID-19 pandemic prevented us from achieving the planned number of clusters and prescriptions per cluster, resulting in reduced statistical power. Notably, the study was conducted in only 4 of mainland China’s 31 provinces, and the results should not be overextrapolated to other regions due to significant variations in guideline awareness and compliance. Future research should include physicians from more provinces. Second, the study focused solely on adherence to ceftriaxone-based guidelines. Given the emergence of new antigonococcal antimicrobials, future training programs should incorporate guidelines for alternative antibiotics. Third, while cluster randomization ensured good balance at the hospital and physician levels, residual confounding or unmeasured contextual factors may have influenced the results. Additionally, the inability to mask physicians to their intervention group introduced Hawthorne effect bias. To mitigate this, we avoided direct observation during prescribing and collected outcome data in either group after the 6-month intervention. Finally, the qualitative component had a small sample size, and the participant sampling process and demographic characteristics were not systematically documented. This methodological gap may limit the generalizability of the findings. Future studies should prioritize multicenter, large-scale designs integrating quantitative and qualitative methods to enhance validity.

### Future Directions

More robust evidence is needed on the efficacy of currently available generic antibiotics for gonorrhea treatment and their resistance surveillance. This evidence should inform the development of local evidence-based guidelines and be incorporated into training programs. With such data supporting the use of generics, physicians can be more confident in prescribing generic ceftriaxone at recommended dosages. Fortunately, postmarket evaluation of generic medicines has been included in the ROADMAP research plan to address gonococcal resistance in China [34]. In 2016, the National Medical Products Administration (NMPA), formally known as the China Food and Drug Administration, mandated that generic drugs approved before 2007 must demonstrate bioequivalence to brand-name counterparts by the end of 2021 [35]. To correct misconceptions about generic drugs, the NMPA should also ensure that generic drugs meet quality standards by using good manufacturing practices and that the generic drug approval process is rigorous and transparent to the public.

Once evidence-based guidelines are established, efforts to reduce inappropriate antibiotic prescriptions can be enhanced by training physicians via electronic platforms. Future research should also explore whether applying training long-term or adding other interventions beyond online training can further optimize antibiotic use for gonorrhea.

### Conclusions

In conclusion, our study demonstrates that a 6-month intervention involving an app-based training tool with monthly reminders to physicians did not significantly improve adherence to the National Guidelines for gonorrhea treatment with ceftriaxone. A key barrier to adherence was physicians’ concerns about the perceived insufficient efficacy of currently available generic ceftriaxone. Addressing these concerns will require postmarket reevaluation of generic ceftriaxone products and targeted education for physicians on the scientific evidence supporting the efficacy of generics. These steps are essential to promote appropriate prescribing behavior.

## Supplementary material

10.2196/63736 Multimedia Appendix 1 Follow-up questionnaire survey for the physicians in the intervention hospitals.

10.2196/63736 Multimedia Appendix 2 Interview outline.

10.2196/63736 Multimedia Appendix 3 Mean compliance rates of antibiotic prescribing for the treatment of gonorrhea per month by (A) different groups and (B) provinces. App-based training intervention introduced at month 0. Error bars indicate 95% CIs.

10.2196/63736 Checklist 1 CONSORT-eHEALTH (V 1.6.1) checklist.

## Abbreviations

AOR: adjusted odds ratio
NMPA: National Medical Products Administration
STD: sexually transmitted disease
