# Supplementary material for: App-Based Training Module on Guiding Physicians’ Prescription for Antibiotic Treatment of Gonorrhea: Cluster Randomized Controlled Trial
Source: JMIR Mhealth Uhealth. 2026 Mar 4;14:e63736. doi: 10.2196/63736 (PMC12978911; doi:10.2196/63736)
Supplement: Multimedia Appendix 1 [file mhealth-v14-e63736-s001.pdf]

Multimedia Appendix 1: Follow-up questionnaire survey for the physicians in the intervention hospitals

This form is used for collecting information after a cluster randomized controlled trials (RCT) is conducted at your hospital. You are invited to participate in this questionnaire survey which takes you about 10 minutes to answer all questions. Participation in this survey is totally voluntary and you can choose either participation to answer all or some questions or rejection to participate. Any choice does not influence the relationship between you and the investigators.
Thank you in advance for your time in considering this questionnaire survey or completing this questionnaire.

1. Your working number:

❍ □□□□ (*Assigned*)

2. Your gender:

❍ Male
❍ Female

3. Your age:

❍ <25
❍ 25~35
❍ 35~45
❍ 45~55
❍ 55~65
❍ >65

4. What is the highest education degree that you have obtained?

❍ Post-Doctor
❍ Doctor
❍ Master
❍ Bachelor
❍ Other (Specific:____)

5. The name of hospital you are working at:

- ________________________________(Text)

6. What department do you work in the hospital?

- Dermatology
- STD
- Andrology
- Urology
- Obstetrics and gynecology
- Other (Specific:____)

7. How long have you been working in this department?

- ≤5 years
- 6~10 years
- ≥11 years

8. Your current professional title is:

- Chief physician
- Associate chief physician
- Physician-in-charge
- Resident physician
- Other (Specific:____)

9. In the last three months, approximately how many cases of uncomplicated gonorrhea have you treated?

- ______________(Number input) (If answer <1, Skip to End of Survey)

10. Before participating in the on-line video-based training, do you know about the up-to-dated National STD Treatment Guidelines for treatment of uncomplicated gonorrhea?

- Yes
- No

11. In the last six months, how many times did you access to the on-line training video developed on the Xieshou platform?

- ❍ 0
- ❍ 1~3
- ❍ 4~6
- ❍ ≥7

12. In the last six months, have you received other relevant trainings on treatment of gonorrhea?

- ❍ Yes (Specific:____)
- ❍ No

13. What do you think about the up-to-dated regimen in the National STD Treatment Guidelines for treatment of uncomplicated gonorrhea using ceftriaxone 1g as a single dose?

- ❍ Appropriate (enough) dosage
- ❍ Dosage is overdose
- ❍ Dosage is not enough
- ❍ Difference from case to case
- ❍ Don't know

14. Do you think this on-line video-based training programme is helpful for improving your knowledge on management of gonorrhea?

- ❍ Very helpful
- ❍ Helpful
- ❍ Not helpful
- ❍ Don't know

15. Do you think the participation in this on-line video-based training programme every two months occupy too much of your time?

- ❍ Yes
- ❍ No

16. Will you continue to use this on-line video-based training programme?

- ❍ Yes
- ❍ No

17. Are you willing to recommend this on-line video-based training programme to other colleagues?

- ❍ Yes
- ❍ No

18. Do you have any suggestions on the on-line training programmes?

- ❍ Yes (Specific:____)
- ❍ No
