# Supplementary material for: App-Based Training Module on Guiding Physicians’ Prescription for Antibiotic Treatment of Gonorrhea: Cluster Randomized Controlled Trial
Source: JMIR Mhealth Uhealth. 2026 Mar 4;14:e63736. doi: 10.2196/63736 (PMC12978911; doi:10.2196/63736)
Supplement: Multimedia Appendix 2 [file mhealth-v14-e63736-s002.pdf]

Multimedia Appendix 2: Interview outline

1. Are you familiar with the hospital's regulations on the management of antibiotic use? Do you think you have strictly followed them?

2. Have you received any related prescription reviews and feedback? Have you ever been punished for improper use of antibiotics? Have these regulatory activities had an impact on your antibiotic prescription behavior? Why or why not?

3. Have you participated in any antibiotic use training organized by the hospital? How often? What do you think of these trainings (content, form, frequency, etc.)? Do these trainings have guiding value for your antibiotic prescription behavior?

4. Do you think the antibiotics currently equipped in the hospital and their usage scope can meet your clinical work needs? Is your income related to the antibiotic prescriptions you issue?

5. What factors do you mainly consider when prescribing antibiotics? For example, following relevant disease diagnosis and treatment guidelines, your own experience, patient needs, drug prices, practices of peers, etc.

6. Before participating in this study, did you receive the latest version of the sexually transmitted disease diagnosis and treatment guidelines? If so, in what form did you receive it (print version, electronic version, etc.)? Do you think the STD diagnosis and treatment guidelines are useful for your clinical practice? If so, why? If not, why not? What's your opinion on the treatment recommendations in the latest STD diagnosis and treatment guidelines, which suggests using a single dose of 1g ceftriaxone for the treatment of gonorrhea?

7. What's your opinion on the training videos in this study? Compared with other forms of training you have received related to the use of antibiotics, how do you think it is? How were you informed to participate in this video training? Under what circumstances do you usually watch the training videos (actively or passively)? What's the frequency? Has this training increased your knowledge in the diagnosis and treatment of gonorrhea? Do you think it is necessary to be reminded regularly and participate in the training multiple times? Does being reminded regularly to participate in the training make you feel uncomfortable? Has this training affected your prescription behavior? Why or why not?

8. What's your opinion on the training platform of this study? Under what circumstances do you usually use the platform? What's the frequency? Have you encountered any problems during the use? Do you have any suggestions?

9. Do you have any suggestions for the future promotion of STD diagnosis and treatment guidelines?
