# Supplementary material for: App-Based Training Module on Guiding Physicians’ Prescription for Antibiotic Treatment of Gonorrhea: Cluster Randomized Controlled Trial
Source: JMIR Mhealth Uhealth. 2026 Mar 4;14:e63736. doi: 10.2196/63736 (PMC12978911; doi:10.2196/63736)

Multimedia Appendix 3: Mean compliance rates of antibiotic prescribing for treatment of gonorrhoea per month by different groups (A) and provinces (B). App-based training intervention introduced at month 0. Error bars indicate 95% CIs.

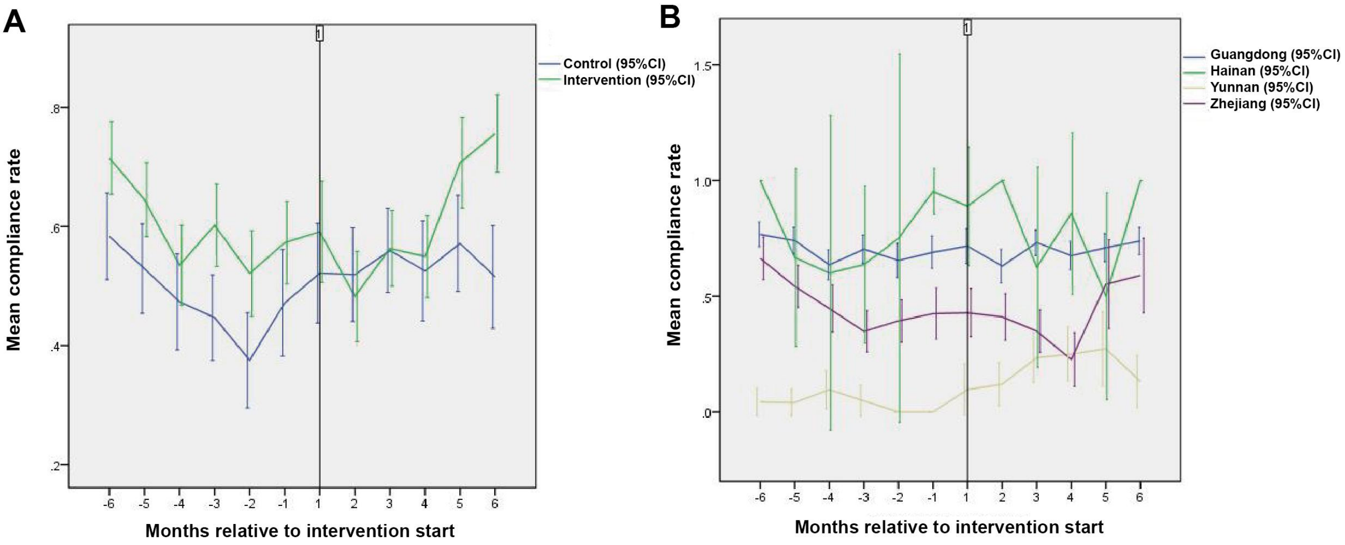

Supplement: Multimedia Appendix 3 [file mhealth-v14-e63736-s003.pdf]
